# Mid-term results of interventional closure of patent foramen ovale with the Occlutech Figulla^®^ Flex II Occluder

**DOI:** 10.1186/s12872-016-0391-3

**Published:** 2016-11-10

**Authors:** Jonas Neuser, Muharrem Akin, Udo Bavendiek, Tibor Kempf, Johann Bauersachs, Julian D. Widder

**Affiliations:** Department of Cardiology und Angiology, Hannover Medical School, Hannover, D-30625 Germany

**Keywords:** Patent foramen ovale, Interventional PFO closure, Occlutech Figulla^®^ Flex II

## Abstract

**Background:**

Patients with a patent foramen ovale (PFO) who suffered from stroke, TIA or peripheral paradoxical embolism are at substantial risk for recurrent neurologic events and in need for secondary prevention. Interventional closure of PFO has been performed for over 20 years. Numerous devices have been developed and used for treatment. We investigated PFO closure with the third generation Occlutech Figulla^®^ Flex II Occluder device.

**Methods:**

Between 2012 and 2015 57 patients (mean age 47.3 ± 1.5 years) who had suffered from a thromboembolic event of unknown cause underwent transcatheter PFO closure with the Occlutech Figulla^®^ Flex II Occluder at our department. 68.4 % of all patients had suffered from cryptogenic stroke, while TIA had occurred in 28.1 %. Almost all patients were diagnosed with an atrial septum aneurysm (90.9 %) and a severe right-to-left shunt grade 3: >20 microbubbles (92.0 %). Follow-up was done 6 months post intervention by clinical examination and transesophageal contrast echocardiography.

**Results:**

No major periprocedural or in-hospital complication occurred. Closure was sufficient with no residual right-to-left shunt in 94.4 % of all patients at 6 months post implantation and only minimal residual shunt in three cases. There were no thrombotic formations associated to the occluder device. Atrial fibrillation occurred in one patient and a recurrent cerebral ischemic event was seen in one patient, who suffered from another TIA.

**Conclusions:**

The Occlutech Figulla^®^ Flex II Occluder device and its delivery system is safe and provides sufficient closure of PFO in patients who suffered from cryptogenic stroke, TIA or paradoxical peripheral embolism.

## Background

**Fig. 1:**
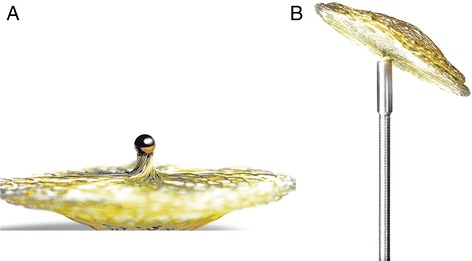
Pictures of the Occlutech Figulla^®^ Flex II. **a** Occlutech Figulla^®^ Flex II ball-socket right atrial hub. **b** Occlutech Figulla^®^ Flex II attached to the delivery system

Paradoxical embolism caused by a patent foramen ovale (PFO) is considered to be an important cause of cryptogenic ischemic stroke especially in young patients [1]. Prevalence of a PFO is up to 40 % in patients with stroke [2]. Patients who suffered from ischemic stroke without determined origin were shown to have larger PFO with more extensive right-to-left interatrial shunting in contrast transesophageal echocardiography than patients with determined cause [3]. Especially patients diagnosed with a combination of PFO and atrial septum aneurysm (ASA) and prior stroke are at substantial risk for a recurrent cerebral ischemia [4, 5]. Therapeutic options for secondary prevention of recurrent stroke in these patients include either conservative medical treatment (oral anticoagulation or platelet inhibition) or interventional closure. Reports about the efficacy of medical treatment to prevent recurrent stroke are diverse [4, 6]. Transcatheter closure of PFO has been shown to be feasible over 20 years ago [7]. Recent cost-effectiveness analysis revealed that medical treatment cost exceeded interventional PFO closure cost at about 30 years post implantation [8]. Over the years numerous devices with different structures and varying anticoagulation or antithrombotic therapy regimes have been used [9–12]. Controversy though still exists about the indication for PFO closure. Three randomized clinical trials of transcatheter device PFO closure versus medical therapy have been published yet [9–11]. All three failed to demonstrate significant superiority of interventional PFO closure over medical management in the intention-to-treat analysis. Per-protocol and as-treated analyses of the RESPECT Trial though indicated superiority of PFO closure vs. medical therapy. Subgroup post hoc analysis of the RESPECT Trial suggest that patients with substantial right-to-left interatrial shunt or ASA seem to profit probably most from PFO closure. As the medical treatment therapy compared to PFO occlusion results in life long antiplatelet or oral anticoagulation therapy, interventional closure is considered reasonable by numerous physicians. Post interventional closure rates differ depending on the device used and have been described between 86 and 96 % [9–11, 13, 14]. The Amplatzer^TM^ PFO Occluder (ST Jude Medical^TM^, Saint Paul, Minnesota, USA) is probable the most widely used occluder device and was recently studied in the two large multi-center RESPECT and PC trials. Other devices like the GORE^®^ HELEX^®^ (W. L. Gore & Associates, Inc, Newark, Delaware, USA), Nit-Occlud^®^ PFO (pfm medical ag, Cologne, Germany), Cardi-O-Fix PFO Occluder (Starway Medical Technology, Inc, Beijing, China) and CeraFlex^TM^ PFO Occluder (Carlsbad, California, USA) are available [9, 11, 14–17]. Some devices such as the STARFlex^®^ (NMT Medical, Inc, Boston, Massachusetts, USA), studied in the CLOSURE I Trial have disappeared from the market [10, 12]. Occlutech (Jena, Germany) launched their first occluder in 2003 and their current third generation occluder device, available since October 2011, is called Figulla^®^ Flex II. The Figulla^®^ PFO devices are build out of a nitinol wire mesh, which is shaped to two discs by a distinctive braiding technique, which allows to reduce material in the left atrium [18]. There are four sizes with diameters 16/18, 23/25, 27/30 and 31/35 mm available. Another unique characteristic of the Figulla^®^ devices is the flexibility of the occluder device, which can be angled independently from the angle of the delivery system due to a ball-socket joint connection. This enables a preferred occluder placement prior to release of the occluder even under difficult anatomic circumstances [19, 20]. Like previous Occlutech Figulla^®^ devices the Figulla Flex II has a single layer left atrial disc and only one right atrial hub (Fig. 1a). Modifications of the delivery system allow now greater flexibility and range of angulation and the left atrial material content is minimized providing a superior adaptation to the interatrial septum (Fig. 1b).

In order to reevaluate our standard practice we retrospectively analyzed data from patients with cryptogenic ischemic stroke, transient ischemic attack (TIA) or paradoxical embolism, which underwent percutaneous closure of PFO with the Figulla^®^ Flex II Occluder device.

## Methods

### Patients

We retrospectively studied consecutive patients with a history of ischemic stroke, TIA or peripheral embolism, who were diagnosed with a PFO and underwent transcatheter PFO closure with the Figulla^®^ Flex II Occluder at our Department between November 2012 to July 2015. Cryptogenic stroke, TIA or paradoxical embolism was diagnosed at the Department of Neurology or by a referring neurologist. Cerebral imaging (computer tomography or magnetic resonance imaging) had been performed in all patients and at least one 12-lead EKG, echocardiography, cardiac monitoring for at least 24 h with automated rhythm detection and imaging of the both extracranial and intracranial arteries had been done.

### Echocardiography

Transesophageal multiplane contrast echocardiography was performed in all patients to diagnose PFO and ASA. For those examinations performed in our department, quantification of the right-to-left shunt was assessed before, during and after Valsalva maneuver, following the graduation of the RESPECT Trial. Numbers of passing contrast microbubbles were counted and categorized according to the following grades: grad 0: no microbubbles, grade 1: 1–9 microbubbles, grade 2: 10–20 microbubbles, grade 3: >20 microbubbles as described earlier [9]. Spontaneous appearance of contrast was considered to be grade 3. ASA was defined by an excursion of the septum of ≥10 mm, following the RESPECT Trial as well [9]. Transesophageal multiplane echocardiography cines from examinations performed by referring centers were reviewed by the echocardiographic specialist from our department as well as by the interventionalist and graduation of the right-to-left shunt rated as described above. For those examinations, which were done in our department, PHILIPS EPIQ 7 or iE33 ultrasound machines were used.

### Implantation procedure

All implantation procedures were performed at the Department of Cardiology and Angiology at Hannover Medical School, Germany. The Occlutech Figulla^®^ Flex II Occluder device and its delivery system have been described earlier [21]. In brief, implantation was performed in local anesthesia and under transesophageal multiplane echocardiography as well as fluoroscopy guidance. After puncture of a femoral vein and passage of the PFO with a 6 F multipurpose catheter (Cordis, Miami, Florida, USA), the multipurpose catheter was changed to a transseptal sheath by using a 0.032 heavy duty wire (COOK Europe, Bjaeverskov, Denmark). The Occlutech transseptal deliver system (8 to 9 F) was then advanced into the right atrium. The distance from the PFO to the aortic root and the distance from the PFO to superior vena cava orifice, as well as the PFO-defect size were measured by transesophageal multiplane echocardiography. Size of the occluder device (23/25 mm vs. 27/30 mm) was chosen depending on echocardiographic findings. After passing the atrial septum, implantation of the occluder was accomplished under echocardiographic and fluoroscopic guidance. If a satisfying device position had been achieved, the delivery system was disconnected from the occluder. Following the implantation correct positioning and sufficient closure were verified by echocardiography with colour-doppler imaging. Successfully closure was considered if no or only minimal residual shunt was detected. The delivery system was removed and manual compression applied on the venous puncture side until hemostasis was achieved. Thereafter patients underwent compression bandage of the access site for 8 h.

### Medical treatment

Patients received 7500 U of heparin intravenously during the implantation procedure. Cefazolin or vancomycin, in cases of known allergy, was used for periprocedural anti-infective prophylaxis. Endocarditis prophylaxis was recommended for at least 6 months post implantation. Dual antiplatelet therapy with aspirin (100 mg/die) and clopidogrel (75 mg/die) was recommended for all patients during at least the first 6 months following the procedure. Patients received a loading dose of clopidogrel (600 mg) the day before the procedure. Before discontinuation of dual antiplatelet therapy thrombus formation on the occluder device was ruled out by transesophageal echocardiography.

### Follow-up

Six months after implantation procedure patients were requested to visit the outpatient clinic and interviewed asking for a recurrent neurologic event or paradoxical embolism, other adverse events such as hospitalization or bleeding as well as occurrence of arrhythmias. Further transesophageal contrast echocardiography was performed, following the baseline examination procedure. Special attention was paid on detection of potential residual shunts and thrombus formation as well as accurate position of the occluder device.

### Statistics

All results are presented as mean ± standard error. Statistical analyses were done using IBM SPSS Statistics (Armonk, New York, US).

## Results

### Patients and echocardiography

**Fig. 2:**
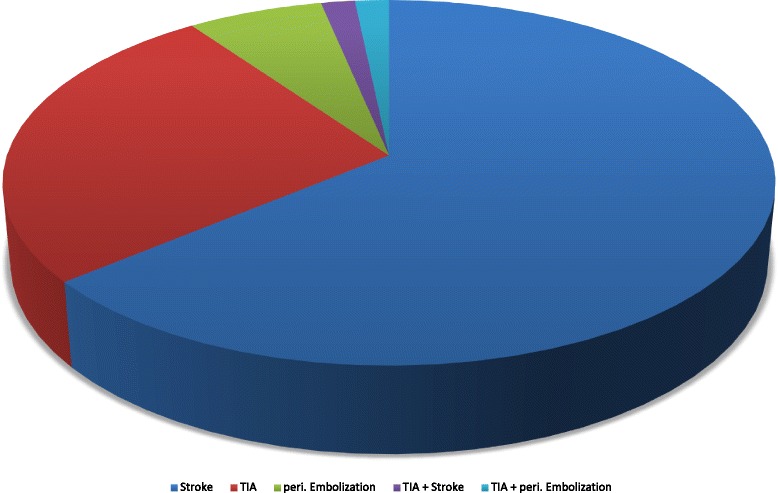
Distribution of type of previous paradoxical embolic event

**Fig. 3:**
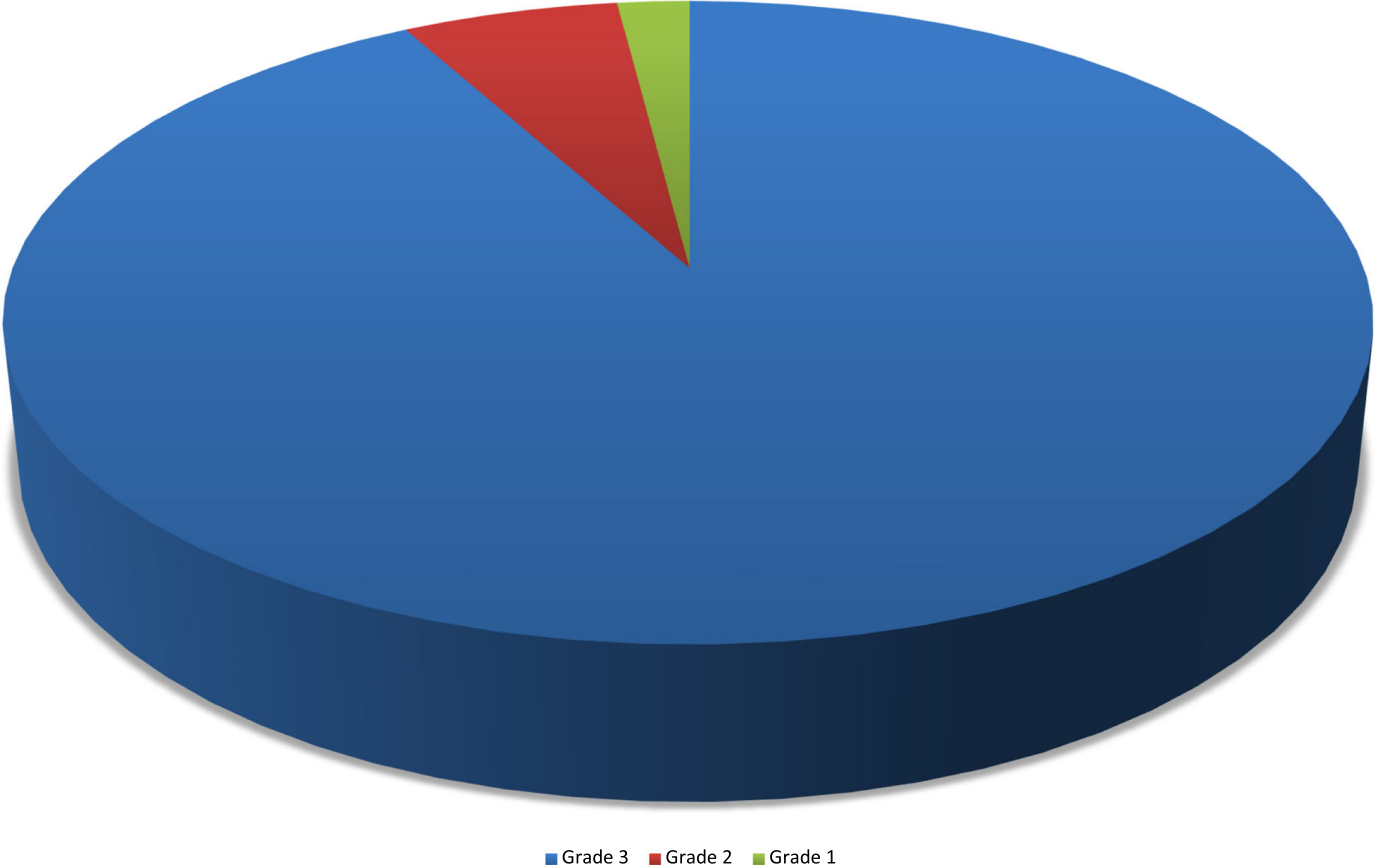
Distribution of right-to-left shunt size before interventional PFO closure

During the period from November 2012 to July 2015 57 consecutive patients who had suffered from at least one thromboembolic event underwent transcatheter PFO closure with the Figulla Flex II Occluder device from Occlutech^®^ at our Department. As shown in Table 1 mean age at implantation was 47.3 ± 1.5 years. Previous stroke had occurred in 68.4 % and TIA in 28.1 % of all patients. Three patients had suffered only from recurrent paradoxical peripheral embolism. Two patients had two previous events, one had a TIA and a stroke while the other patient had a history of stroke as well as of paradoxical peripheral embolism in the right brachial artery (Fig. 2). Nine out of ten patients presented with a coexisting ASA (90.9 %) and a grade 3 right-to-left shunt (92.0 %) (Fig. 3).

**Table 1:** Baseline Characteristics

Characteristic	
Age (yrs)	47.3 ± 1.5
Gender (male sex)	32/57 (56.1 %)
Body-mass index (kg/m^2^)	26.4 ± 0.8
Arterial Hypertension	23/57 (40.4 %)
Diabetes mellitus	4/57 (7.0 %)
Hypercholesterolemia	24/57 (42.1 %)
Cerebrovascular Event	
- TIA	16/57 (28.1 %)
- Stroke	39/57 (68.4 %)
Peripheral embolism	4/57 (7.0 %)
Atrial septum aneurysm	50/55 (90.9 %) ^a^
Significant interatrial right-to-left-shunt	46/50 (92.0 %) ^b^

Values are given n (%), except age and body-mass index given as mean ± SEM, no record in ^a^2, ^b^7 patients

### Implantation procedure

Device implantation was successfully performed in all patients. Occluder device sizes used were: 23/25 mm in 51 (89.5 %) and 27/30 mm in six patients (10.5 %). No major procedure-related complications occurred during the implantation.

### Follow-Up

Complete follow-up data was available for 54 patients. Mean hospital stay was 1.5 ± 0.09 days. No major in-hospital complication took place in any case and no post-procedural AV-fistula, aneurysm or thrombosis was detected.

During the first 6 months following implantation procedure one patient (1/57, 1.75 %) was diagnosed with a recurrent neurologic event. Even though sufficient closure with neither residual right-to-left shunt nor thrombus formation was diagnosed by transesophageal contrast echocardiography, he suffered most likely from another TIA. The patient, a 48-year old male, current cigarette smoker presented with paresthesia in his left fingertips, 3 months after percutaneous PFO closure. Cranial magnetic resonance imaging, cranial computer tomography as well as standard electroencephalography and electroneurographic examination did not show any pathologic findings. Eventually the event was seen as a suspicious case of a TIA by an experienced neurologist.

**Fig. 4:**
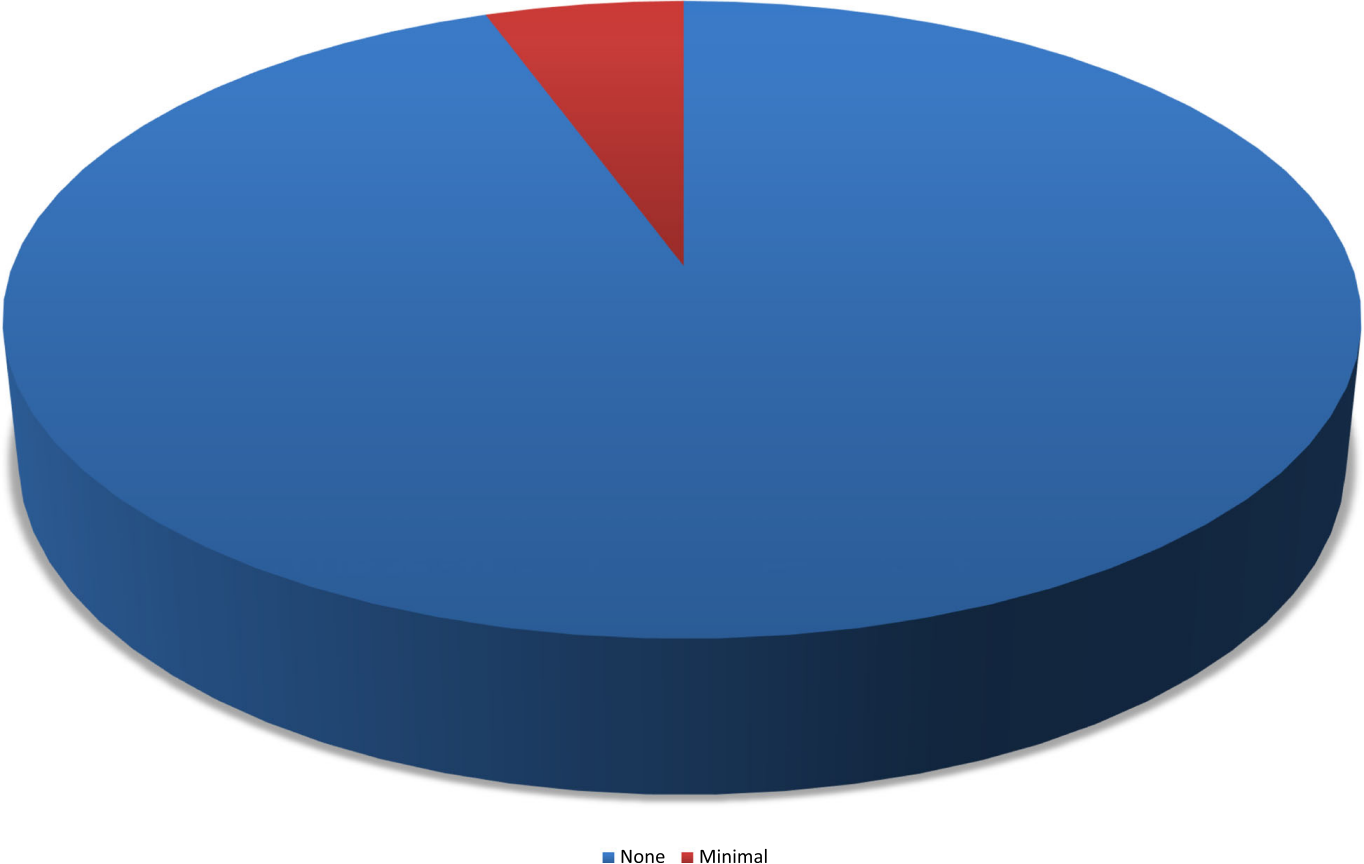
Distribution of right-to-left shunt 6 months after interventional PFO closure

Follow-up echocardiography excluded thrombus formation in all other cases as well. Persistent right-to-left shunt was apparent in three patients, even though quantification revealed only minimal residual shunt in all three cases (Fig. 4). One patient, a 69-year-old female, who had undergone closure following explicit patient’s wish, was diagnosed with paroxysmal atrial fibrillation and antithrombotic therapy was switched to oral anticoagulation.

## Discussion

Our retrospective single-center study shows the Figulla^®^ Flex II Occluder from Occlutech to be a safe and reliable device for interventional PFO closure. Few studies on PFO closure were performed using the Occlutech Figulla^®^ devices, and to our knowledge no reports on the third generation Figulla^®^ Flex II PFO Occluder are available [15, 18–20, 22–24].

There were no major periprocedural or in-hospital complications, especially no vascular complications such as AV fistula, thrombosis or aneurysm after puncture of a femoral vein and patients were dismissed within the first 4 days after implantation procedure. Other formerly reported complications such as device embolization or intraprocedural TIA did not occur as well [20]. All patients were initially treated with the same combination of anti-platelet therapy in form of aspirin 100 mg/die and clopidogrel 75 mg/die.

Complete data from transesophageal contrast echocardiography, performed 6 months post implantation, was available in 94.7 % of all cases. Examinations did not reveal any thrombus formation. Minimal residual right-to-left shunting was seen in only three cases and did not require intervention. Considering sufficient closure to be defined as no or only minimal residual right-to-left shunting, we were able to reach high sufficient closure rates using the Figulla^®^ Flex II PFO Occluder when comparing our results to former studies: Using the Amplatzer^TM^ device Maier et al. reported sufficient closure in 95.9 % in the PC Trial and Carroll et al. 93.5 % in the RESPECT Trial, while sufficient closure was achieved in only 86.1 % in the CLOSURE I Trial studying the STARFlex^®^ device [9–11]. In previous studies using earlier generation Figulla^®^ PFO devices closure rates between 83 and 97 % were reached [18, 20, 23].

Only one patient was diagnosed with atrial fibrillation within the first 6 months after device implantation. However, this female patient was 69 years of age. Prevalence of atrial fibrillation is known to increase with age and was shown to be up to 2.7 % in female patients at that age [25, 26]. Considering this data a clear dependency of the new onset atrial fibrillation and the device implantation is unlikely. In all other cases antithrombotic therapy was discontinued after the period of 6 months post closure procedure, as long as no other indication for an anti-platelet treatment was apparent.

One recurrent neurologic event within the first 6 months following PFO closure occurred. The occluder device provided sufficient closure and thrombus formation could be precluded by transesophageal contrast echocardiography. Since further examinations did not reveal pathologies, the reported symptoms of the 48-year old male patient, a current smoker, were interpreted as a suspicious case of a TIA. However, a direct association to the occluder device is questionable. Furthermore, the event rate is in range of former studies with different occluder devices as listed in Table 2. Using the Amplatzer^TM^ device Carroll et al. and Maier et al. reported recurrent neurologic events in 3 % of all PFO closures in the RESPECT Trial respectively PC Trial, while recurrent neurologic events occurred in 6 % after PFO closure with the STARFlex^®^ in the CLOSURE I Trial [9–11]. Similar results were reported in a study, which was performed at our department, using the Amplatzer^TM^ device [27]. Though, the shorter follow-up period in our present study must be considered.

**Table 2:** Recurrent Neurologic Event Rate

Study	TIA	Ischemic Stroke	Follow-Up Period
RESPECT Trial (Amplatzer^TM^)^9^	6/499 (1.2 %)	9/499 (1.8 %)	2.6 years (mean)
PC Trial (Amplatzer^TM^)^11^	5/204 (2.5 %)	1/204 (0.5 %)	4.1 years (mean)
CLOSURE I Trial (STARFlex^®^)^10^	13/447 (3.1 %)^a^	12/447 (2.9)^a^	2 years
Fischer et al. (Amplatzer^TM^)^27^	2/113 (1.8 %)	1/113 (0.9 %)	17.7 months (mean)
Krizianic et al. (Figulla^®^ N)^23^	0/34 (0 %)	1/34 (2.9 %)	6 months
Aytemir et al. (Figulla^®^)^19^	1/85 (1.2 %)	0/85 (0 %)	17.0 months (mean)

^a^Intention-to-treat-population

Since almost all patients presented with a coexisting ASA conclusions are limited to this patient subgroup. Former studies revealed that especially these patients are at risk for a recurrent cerebral ischemia [4, 5]. The RESPECT Trial showed a favorable outcome of percutaneous PFO closure in patients with ASA and substantial right-to-left interatrial shunt [9]. The main limitations of our study are its retrospective design and the relatively short observation period as well as the fact that three patients had refused TEE follow up, however a telephone interview was performed and no event reported.

## Conclusions

In conclusion, the Figulla^®^ Flex II PFO Occluder and its delivery system are safe and provide sufficient closure of intra-atrial communication in patients with PFO and ASA, who had suffered from cryptogenic stroke, TIA or paradoxical peripheral embolism, comparable to former studies with the Amplatzer^TM^ Occluder systems.

## Abbreviations

ASA: Atrial septum aneurysm
PFO: Patent foramen ovale
TIA: Transient ischemic attack
